# The Effects of Cadmium at Low Environmental Concentrations on THP-1 Macrophage Apoptosis

**DOI:** 10.3390/ijms160921410

**Published:** 2015-09-07

**Authors:** Tomasz Olszowski, Irena Baranowska-Bosiacka, Izabela Gutowska, Katarzyna Piotrowska, Katarzyna Mierzejewska, Jan Korbecki, Mateusz Kurzawski, Maciej Tarnowski, Dariusz Chlubek

**Affiliations:** 1 Department of Hygiene and Epidemiology, Pomeranian Medical University, Szczecin 70-111, Poland; E-Mail: tomasz.olszowski@pum.edu.pl; 2 Department of Biochemistry and Medical Chemistry, Pomeranian Medical University, Szczecin 70-111, Poland; E-Mails: jan.korbecki@onet.eu (J.K.); dchlubek@sci.pam.szczecin.pl (D.C.); 3 Department of Biochemistry and Human Nutrition, Pomeranian Medical University, Szczecin 71-460, Poland; E-Mail: izagut@poczta.onet.pl; 4 Department of Physiology, Pomeranian Medical University, Szczecin 70-111, Poland; E-Mails: piot.kata@gmail.com (K.P.); kasia.mierzejewska@gmail.com (K.M.); maciejt@sci.pum.edu.pl (M.T.); 5 Department of Experimental and Clinical Pharmacology, Pomeranian Medical University, Szczecin 70-111, Poland; E-Mail: mkurz@op.pl

**Keywords:** cadmium, apoptosis, ROS, mitochondrial membrane potential, Bax, Bcl-2, THP-1 macrophages

## Abstract

Cadmium at environmental concentrations is a risk factor for many diseases, including cardiovascular and neurodegenerative diseases, in which macrophages play an important role. The aim of this study was to evaluate the effects of cadmium at low environmental (nanomolar) concentrations on apoptotic processes in THP-1(acute monocytic leukemia cells line)-derived macrophages, with special focus on mitochondrial events involved. Macrophages were incubated with various cadmium chloride (CdCl_2_) solutions for 48 h at final concentrations of 5 nM, 20 nM, 200 nM and 2 µM CdCl_2_. Cell viability was measured using flow cytometry. Flow cytometric measurement (annexin V/FITC (annexin V/fluorescein isothiocyanate) and PI (propidium iodide) double staining) was used to quantify the extent of apoptosis. Fluorescence and confocal microscopy were used for imaging of apoptosis process. Changes in mitochondrial membrane potential were monitored using cytofluorimetry after cell staining with JC-1(5,5′,6,6′-tetrachloro-1,1′,3,3′-tetraethylbenzimidazol-carbocyane iodide) probe. Mitochondrial ROS (reactive oxygen species) levels were measured cytofluorimetrically after incubation of cells with mitochondrial superoxide indicator (MitoSOX) red fluorescent marker. The mRNA expression of *Bcl-2* and *Bax* was analysed with qRT-PCR. Our study demonstrates that cadmium, even at low environmental concentrations, exerts mitochondrial toxicity in THP-1 macrophages. Forty-eight-hour exposure to very low concentrations reduces cell viability and results in cell death by apoptosis and necrosis. The decrease in mitochondrial membrane potential, increased ROS production, increased *Bax* and decreased *Bcl-2* mRNA expression are mitochondrial events involved in cadmium-induced apoptosis.

## 1. Introduction

Cadmium is a toxic, mutagenic and carcinogenic heavy metal constituting substantial threat to public health [1,2,3]. Cadmium is ranked on the 7th place in the 2013 substance priority list created by the agency for toxic substances and disease registry (ATSDR) [4]. The sources of environmental cadmium exposure include diet and tobacco smoking [2,3]. Cadmium affects several organ systems, including respiratory system, kidney, skeletal system and reproductive system [1,3]. Cadmium was suggested to cause atherosclerosis, the basis of most cardiovascular diseases [5].

A number of studies have suggested that cadmium induces apoptosis in several cell types [6,7,8,9,10,11,12,13,14,15,16,17,18,19], including monocytes and macrophages [20,21,22,23,24,25,26,27,28]. Different apoptotic pathways may be induced by cadmium in different cell types and depending on the cell treatment conditions [6]. The mitochondria are thought to be the primary target in cadmium-induced apoptosis [11]. The mitochondrial events suggested to be involved in Cd-induced apoptosis include: increased permeability of the mitochondrial membrane, decreased mitochondrial transmembrane potential, release of cytochrome c and apoptosis-inducing factor (AIF), and production of reactive oxygen species (ROS) [13]. A very important role in the regulation of these events is played by pro-apoptotic (Bax, Bak, Bid, Bim, Bad, Noxa) and anti-apoptotic (Bcl-2, Bcl-x_L_, Mcl-1) members of the Bcl-2 family [29].

Apoptosis, known as programmed cell death, is characterized by distinct morphological characteristics, such as cell shrinkage and convolution, pyknosis and karyorrhexis, intact cell membrane, lack of inflammation and finally, the breakdown of the cell into apoptotic bodies followed by secondary necrosis [6,30]. Apoptosis is induced by two major pathways: the extrinsic (or death receptor) pathway and the intrinsic (or mitochondrial) pathway. The extrinsic signaling pathways that initiate apoptosis involve death receptors that are members of the tumor necrosis factor (TNF) receptor gene superfamily, while the intrinsic signaling pathways involve non-receptor-mediated stimuli producing intracellular signals that act directly on targets within the cell and are mitochondrial-initiated events [30]. A number of stimuli (factors) may induce the intrinsic pathway of apoptosis, among them are heavy metals [31].

Macrophages are a very diverse group of cells that, due to the presence in different organs, play different roles. This group of cells includes the microglia, which are present in the central nervous system [32,33]. Therefore, acute monocytic leukemia cells line (THP-1) macrophages may constitute a simplified/approximated experimental model to study the impact of cadmium compounds on microglial dysfunction. First of all, microglial dysfunction increases the severity of symptoms and accelerates the progress of age-related neurodegenerative diseases, such as Alzheimer’s disease [34]. Besides, THP-1 macrophages may constitute a good experimental model to study novel functions and mechanisms of macrophages in the cardiovascular diseases, including atherosclerosis [35]. Some studies show the association of cadmium with cardiovascular mortality and increased incidence of cardiovascular disease [36,37]; other reports suggest cadmium to be a possible etiological factor for neurodegenerative diseases, such as Alzheimer’s and Parkinson’s diseases [38,39].

Previously, we evaluated the effect of cadmium on cyclooxygenase 1 (COX-1) and cyclooxygenase 2 (COX-2) gene and protein expression, and enzymatic activity in THP-1 macrophages [40]. The aim of our current study was to assess the effect of cadmium at low concentrations (relevant to human blood cadmium levels due to chronic occupational and/or environmental exposure to this metal) on apoptotic processes in THP-1 macrophages, with special focus on mitochondrial events involved.

The concentrations of cadmium and exposure duration applied in the present study are rarely examined in terms of the effects on apoptosis. In the context of cancer progression, regarding competition dynamics between genetically stable and unstable populations, the efficacy of apoptosis influences the conditions under which genetic instability is selected for. Even small changes in apoptosis induced by environmental factors (such as cadmium) might influence genetic instability [41]. Therefore, the results of our study may help to better understand the mechanisms of cadmium toxicity.

## 2. Results

### 2.1. Cadmium and Cell Viability

**Figure 1:**
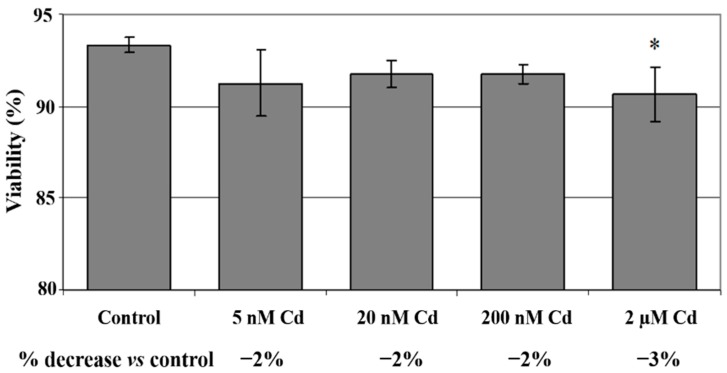
The effect of cadmium on viability of cells cultured with various cadmium chloride solutions. Viability was measured by PI (propidium iodide) incorporation. Viable, alive cells are PI negative, while late apoptotic and dead cells are PI positive. THP-1-derived macrophages were cultured with CdCl_2_ solutions for 48 h. After incubation cells were harvested by scraping and cells viability was measured by flow cytometry analysis. Experiments were conducted as six separate assays (each assay in three replicates). *, statistically significant differences were in comparison to control (*p* < 0.05); Control, cells incubated in roswell park memorial institute (RPMI) 1640 medium with 10% fetal bovine serum (FBS) and H_2_O as vehicle.

THP-1 macrophages were exposed to varying concentrations of cadmium chloride (5 nM, 20 nM, 200 nM and 2 μM) for 48 h. It appeared that THP-1 macrophage viability decreased with increasing cadmium concentration, however the significant reduction in cell viability was observed at the highest applied cadmium concentration, *i.e.*, 2 μM Cd (3% reduction *vs.* control, *p* < 0.05) (Figure 1).

### 2.2. Effects of Cadmium on Early and Late Apoptosis/Necrosis: Imaging of the Apoptosis Process

**Figure 2:**
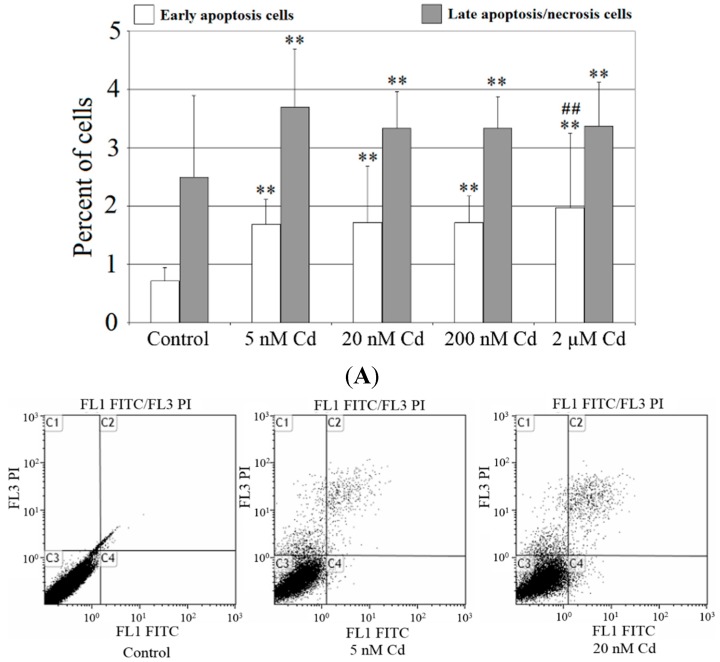

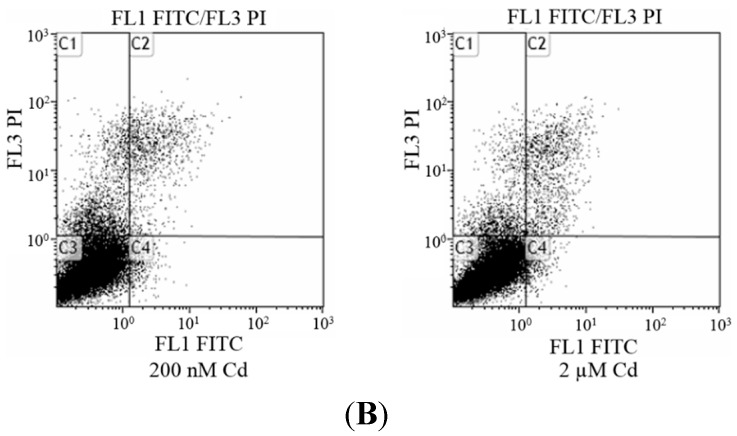
The effect of cadmium on apoptosis in cells cultured with various cadmium chloride solutions. (**A**) THP-1-derived macrophages were cultured with CdCl_2_ solutions for 48 h. After incubation cells were harvested by scraping then were incubated with Annexin V-FITC (1 ng/mL) and propidium iodide (5 ng/mL) for 30 min in the dark and were analysed by flow cytometry. Results are expressed in percentage of apoptotic cells from *n* = six separate experiments; and (**B**) **Lower left** quadrants shows viable cells; **Lower right** quadrants, early apoptotic cells; **Upper left** quadrants necrotic cells; **Upper right** quadrants, nonviable late apoptotic cells. Experiments were conducted as six separate assays (each assay in three replicates). Diagram of representative samples. **, statistically significant differences in comparison to control (*p* ≤ 0.005); ^##^, statistically significant differences in comparison to 5 nM Cd (*p* ≤ 0.005); Control, cells incubated in RPMI 1640 medium with 10% FBS and H_2_O as vehicle.

In this study, flow cytometric measurement (Annexin V/FITC and PI double staining) was used to quantify the extent of apoptosis in the total cell population. Forty-eight-hour exposure to cadmium caused significant, dose-dependent increase in the percentage of early apoptotic cells (FITC^+^, PI^−^) from 135% relative to control (for 5 nM Cd) to 174% relative to control (for 2 μM Cd) (Figure 2A). With the highest cadmium concentration, the early apoptotic cell number rose to approximately 2% (*p* < 0.005) from about 0.7% in control cells (Figure 2B, lower right quadrant).

Forty-eight-hour incubation of THP-1 macrophages with cadmium resulted in significant increase in the percentage of late apoptotic/necrotic cells (FITC^+^, PI^+^) compared to control, with the highest increase for 5 nM Cd (48%). With 5 nM Cd the late apoptotic/necrotic cell number rose to approximately 3.8% (*p* < 0.005) from 2.5% in control cells (Figure 2B).

**Figure 3:**
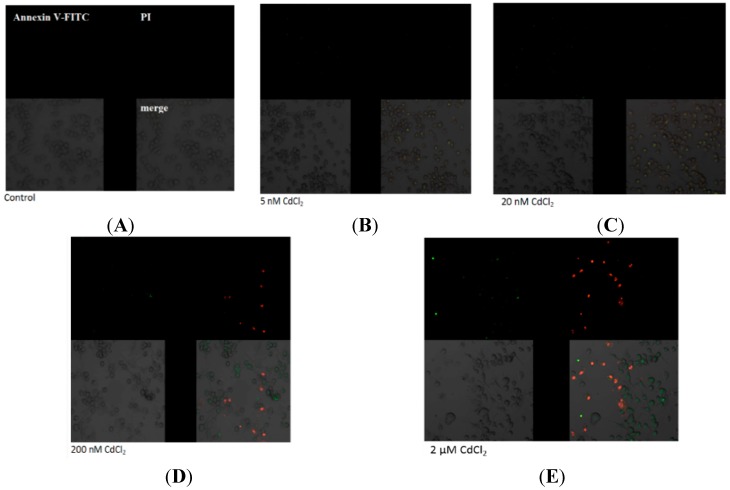
Imaging of apoptosis process by confocal microscopy in macrophages cultured with (**A**) control, cells incubated in RPMI 1640 medium with 10% FBS and H_2_O as vehicle; (**B**) 5 nM CdCl_2_; (**C**) 20 nM CdCl_2_; (**D**) 200 nM CdCl_2_; and (**E**) 2 µM CdCl_2_. Next cells were incubated with Annexin V-FITC (1 ng/mL) and propidium iodide (5 ng/mL) for 30 min in the dark. THP-1 macrophages were cultured with CdCl_2_ for 48 h as described in Section 2. Cells that are viable are Annexin V-FITC and PI negative; in early apoptosis are Annexin V-FITC positive and PI negative (green), in late apoptosis or already dead are both Annexin V-FITC and PI positive (red). A dual-pass FITC/rhodamine filter set was applied.

Figure 3 presents the imaging of apoptosis by confocal microscopy in macrophages treated with various concentrations of cadmium. The Annexin V assay discriminates viable cells (FITC^−^, PI^−^), apoptotic cells (FITC^+^, PI^−^) or necrotic cells (FITC^+^, PI^+^). The results presented here are consistent with the results obtained in Figure 2.

### 2.3. Cadmium Effects on the Expression of Genes Relevant to Apoptotic Process

**Figure 4:**
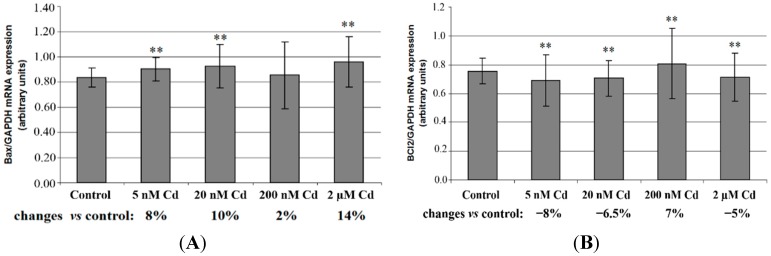

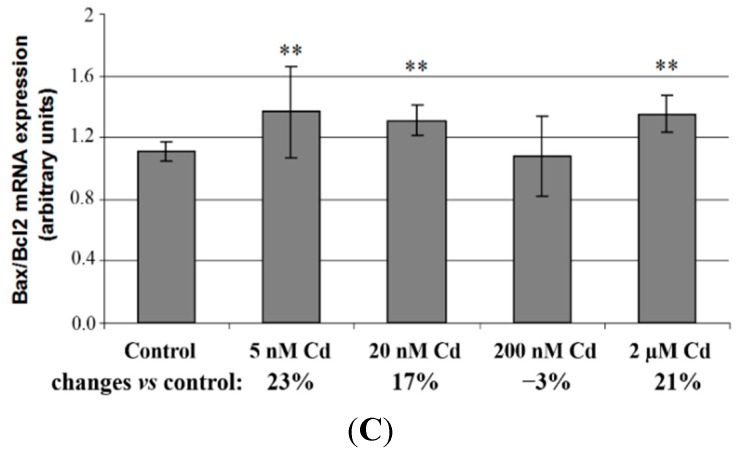
*Bax* (**A**), *Bcl-2* (**B**) and *Bax/Bcl-2* ratio (**C**) mRNA expression in macrophages cultured with various cadmium solutions. Macrophages were cultured with CdCl_2_ solutions for 48 h. After incubation, cells were harvested by scraping and mRNA was measured by real-time PCR method. **, statistically significant as compared with 0 nM Cd-cells incubated in RPMI 1640 medium with 10% FBS and H_2_O as vehicle. Experiments were conducted as six separate assays (each assay in three replicates).

Figure 4 shows *Bax* and *Bcl-2* mRNA relative expression and *Bax*/*Bcl-2* mRNA ratio in macrophages exposed to various concentration of cadmium. Cadmium increased the expression of pro-apoptotic *Bax* mRNA (significant increase for 5 nM, 20 nM and 2 μM Cd in 8%, 10% and 14%, respectively) (Figure 4A) and decreased the expression of anti-apoptotic protein *Bcl-2* mRNA (significant decrease for 5 nM, 20 nM and 2 μM Cd in 8%, 6%, 5% and 5% respectively). As a result, *Bax*/*Bcl-2* mRNA ratio was significantly increased by cadmium exposure (23% increase for 5 nM Cd, 17% increase for 20 nM Cd, and 21% increase for 2 μM Cd). Only 200 nM Cd resulted in insignificant change in *Bax*/*Bcl-2* mRNA ratio (Figure 4C). Thus cadmium chloride treatment significantly shifted the *Bax*/*Bcl-2* mRNA ratio in favor of *Bax*.

### 2.4. Cadmium and Mitochondrial ROS Production

**Figure 5:**
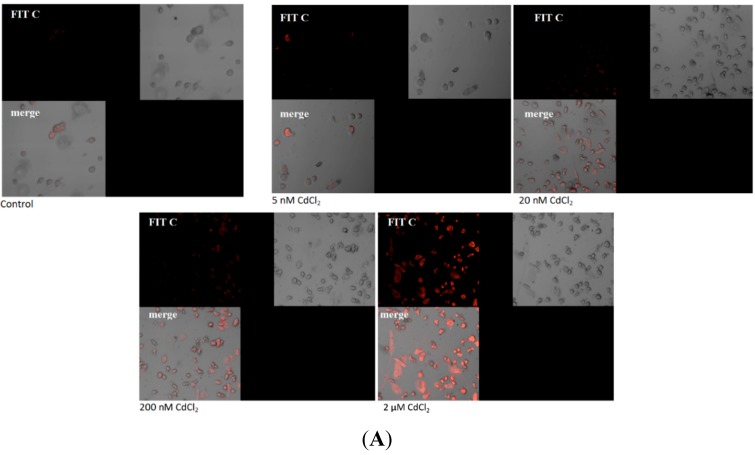

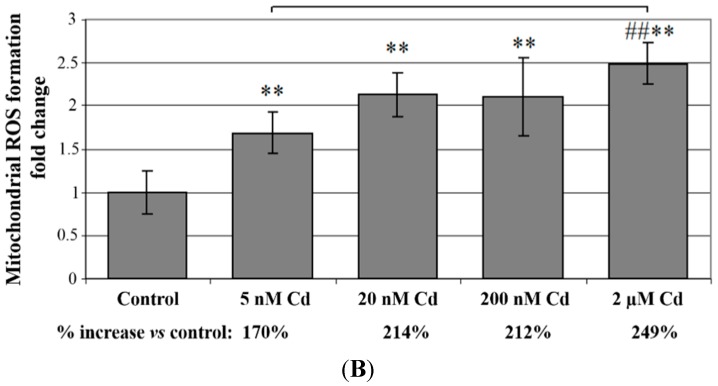
The effect of cadmium on mitochondrial ROS generation in cells cultured with various cadmium chloride solutions. (**A**) Monocytes/macrophages were cultured with CdCl_2_ solutions for 48 h. After, cells were incubated with MitoSOX Red probe for 30 min in the dark, at 37 °C. Cadmium treated cells showed more bright red fluorescence due to the presence of excessive superoxide synthesis in mitochondria; and (**B**) Quantitative measurements of the fluorescence intensity performed by plate reader, normalized to protein content and expressed as relative fold change to the mean value in the control group. The intensity of red fluorescence of Cd-treated cells were significantly higher with respect to control cells in a dose dependent manner. Experiments were conducted as six separate assays (each assay in three replicates) with similar results, thus pictures are representative fields. **, statistically significant differences in comparison to control (*p* ≤ 0.005); ^##^, statistically significant differences in comparison to 5 nM Cd (*p* ≤ 0.005); Control, cells incubated in RPMI 1640 medium with 10% FBS and with H_2_O as vehicle.

Incubation of THP-1-derived macrophages with Mito SOX Red probe revealed more bright red fluorescence in cadmium-treated cells compared to controls due to excessive superoxide synthesis in mitochondria (Figure 5A). The intensity of red fluorescence of cadmium-treated cells (representing mitochondrial ROS formation) was significantly higher with respect to control cells in a dose-dependent manner (Figure 5B). Cadmium caused approximately 1.6–2.5-fold increase in mitochondrial ROS production (Figure 5B).

### 2.5. Cadmium and Mitochondrial Membrane Potential

**Figure 6:**
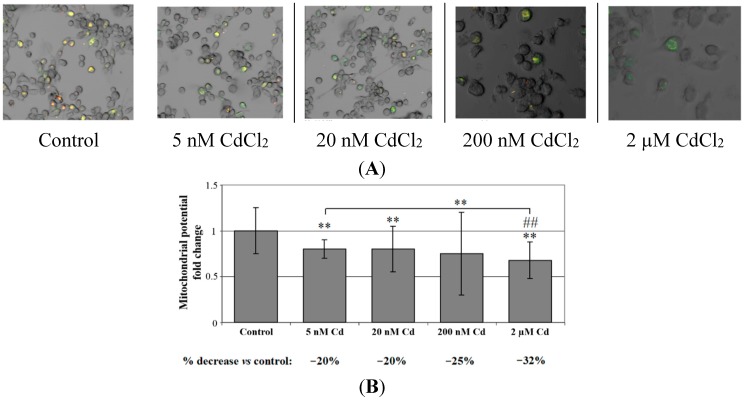
The effect of cadmium on mitochondrial potential (ΨΔm) in cells cultured with various cadmium chloride solutions. (**A**) Monocytes/macrophages were cultured with CdCl_2_ solutions for 48 h. After, cells were incubated with JC-1 probe for 30 min in the dark, at 37 °C. Control cells showed mainly an intense red-orange fluorescence coming from JC-1 aggregates. In contrast, some Cd-treated cells showed also green fluorescence coming from JC-1 monomers; (**B**) Quantitative measurements of the fluorescence intensity performed by plate reader, normalized to protein content and expressed as relative fold change to the mean value in the control group. The intensity of red-orange fluorescence in the Cd-treated cells was significantly lower with respect to control and in dose-dependent manner. Experiments were conducted as six separate assays (each assay in three replicates) with similar results, thus pictures are representative fields. **, statistically significant differences in comparison to control (*p* ≤ 0.005); ^##^, statistically significant differences in comparison to 5nM Cd (*p* ≤ 0.005). Control, cells incubated in RPMI 1640 medium with 10% FBS and with H_2_O as vehicle.

The cells were monitored using mitochondria-specific probe JC-1 to evaluate changes in mitochondrial membrane potential after 48 h of cadmium incubation. As shown in Figure 6A, control cells showed mainly an intense red-orange fluorescence coming from JC-1 aggregates. In contrast, some cadmium-treated cells exhibited also green fluorescence coming from JC-1 monomers. Quantitative measurement of fluorescence intensity revealed that intensity of red-orange fluorescence in the cadmium treated cells was significantly lower with respect to control and in a dose-dependent manner, indicating cadmium-induced dose-dependent decrease in mitochondrial membrane potential after 48 h of exposure to this metal. Cadmium caused approximately 20%–32% decrease in mitochondrial membrane potential (Figure 6B).

## 3. Discussion

The present study examines the *in vitro* induction of macrophage apoptosis and necrosis by cadmium chloride. In our experiments much lower cadmium concentrations were used compared to those applied by other research groups [7,20,21,23,25,26,27]. The cadmium concentrations applied in our study were comparable to blood cadmium levels detected in people chronically exposed to this metal [2]. Therefore, our study is one of the first studies that investigate *in vitro* effects of cadmium compounds that simulate (mimic) human chronic exposure to this metal.

The highest cadmium concentration, 2 μM Cd caused significant reduction in macrophage viability. This is in agreement with the results obtained by Lemarie *et al*., who found that cadmium significantly reduced Hep3B cells viability at concentrations higher than 1 μM Cd after 48 h incubation [10]. In terms of cell viability, THP-1 macrophages seem to be similarly sensitive to cadmium compared to other cells or cell lines, such as U937 cells [20], human peripheral blood lymphocytes [7], human embryonic kidney (HEK) 293 cells [6], human retinal pigment epithelial cells (ARPE-19) [16], human B-cell line Raji and human T-cell line CCRF-CEM cells [15].

In our study, cadmium chloride, even at the lowest concentrations (in the nanomolar range), causes apoptosis and necrosis of THP-1 macrophages after 48 h incubation. This effect is in agreement with the results obtained by other research groups. Cadmium compounds, at the concentration of several micromoles, after incubation period of about 2–12 h, result in apoptosis of U937 monocytes [20,21,22,26,27,28]. After longer incubation period cadmium chloride exhibits similar effect at much lower concentrations in mouse macrophages [23,25,42]. For example, the experiment carried out by Martin *et al.* shows that mouse bone-derived macrophages exhibit apoptosis and significant reduction of viability in submicromolar concentrations of cadmium chloride after 72 h incubation [42]. Nevertheless, one should take into consideration that there may be differences in the response to cadmium between different cell types [43]. Another factor that may contribute to different response to cadmium is experimental conditions (for example different exposure time). Moreover, the hormesis effect observed in certain cells type (for example, in human embryo lung fibroblasts) [44] at low doses of cadmium may not be observed in another cell type (for example, in THP-1 macrophages).

### 3.1. Cadmium and Mitochondria

The intrinsic pathway of apoptosis (mitochondria-dependent) involves the release of pro-apoptotic proteins from mitochondria to cytoplasm. This process is caused by a number of factors. In fact, mitochondria are thought to be the primary target of cadmium-induced apoptosis [9,11].

In our study, exposure to cadmium chloride resulted in the decrease in mitochondrial membrane potential and increase in ROS generated by mitochondria. The obtained results are consistent with the results of other studies *in vitro* [6,8,9,10,13,18,20,24,25,45] and *in vivo* [12]. Cadmium causes disturbances in functioning of complex III of the electron transfer chain resulting in the increase in ROS generation in mitochondria and thus oxidative stress [45,46,47]. Cadmium, through binding the inner mitochondrial membrane, enhances lipid peroxidation and disturbs the integrity of mitochondrial membrane and, as a result, the damage of that organelle [47]. Also, cadmium causes the opening of permeability transition pores [48,49]. As a consequence of those processes the decreased membrane potential and increased ROS generation by mitochondria were observed. The increased ROS level results in the disruption of cell structures and thus cell necrosis [50].

As regards the effects of cadmium on antioxidant defense mechanisms, based on the other studies’ results [51], we might speculate that the results of our study seem to suggest the downregulation of antioxidant defense mechanisms by cadmium, although inconsistent results can be found in the literature. Waisberg and colleagues in their review conclude that “short-term exposure to cadmium has been shown to decrease the activities of almost all of cellular antioxidant enzymes *in vitro* and *in vivo*, whereas with more elevated doses and extended exposure also enhancement of activities was found, probably because of adaptive induction of genes”. [51]. Cadmium can decrease the cellular glutathione content or the activities of superoxide dismutase, glutathione peroxidase and catalase [51]. On the other hand, under certain experimental conditions, cadmium can also stimulate the expression of many genes including those which code for antioxidant proteins [51]. Hsiao and Stapleton exposed primary rat hepatocyte cultures to 4 μM Cd for 3 h and observed a host of genes up-regulated by cadmium that are involved in the synthesis of GSH [52]. Yamada and Koizumi examined changes in the gene expression profile of HeLa cells after exposure to 5 μM CdSO_4_ for 6 h. The authors found that cadmium induced the expression of several antioxidant genes. The cellular metabolism inclined toward the synthesis of cysteine and glutathione after Cd exposure [53].

Since cadmium chloride in our study, apart from apoptosis induction, induced also necrosis, one may not exclude that signaling events observed by us are also part of programmed necrosis called necroptosis where mitochondria also play a role [54]. However, some molecular events observed, such as phosphatidyl serine exposure on the outer leaflet of the plasma membrane plus the results of our previous study demonstrating that cadmium chloride at the concentrations 5 nM–2 µM did not increase the production of some pro-inflammatory factors (COX-2, PGE_2_, TXB_2_) by THP-1 macrophages [40] suggest that the major mode of cell death by cadmium is apoptosis and the observed signaling events refer mainly to apoptosis.

### 3.2. Cadmium and Expression of Pro-Apoptotic Bax and Anti-Apoptotic Bcl-2 mRNAs

As mentioned previously, apoptosis may be induced by the release of pro-apoptotic proteins from the mitochondria to cytoplasm. The Bcl-2 family of proteins is responsible for the regulation of this process [29,55]. This family comprises pro-apoptotic proteins, such as Bax, which are responsible for the formation of pores in mitochondrial membrane by which the pro-apoptotic proteins are released into the cytosol. The Bcl-2 family also consists of the anti-apoptotic proteins, such as Bcl-2, which bind pro-apoptotic Bax protein. Such complexes inactivate pro-apoptotic properties of Bax.

Changes in the expression of Bax and Bcl-2 proteins may influence apoptosis in THP-1 macrophages treated with cadmium chloride. In our study we demonstrated that cadmium chloride at nanomolar concentrations decreases the expression of *Bcl-2* mRNA and increases the expression of *Bax* mRNA. Our results are consistent with the other studies’ results carried out on different experimental models [6,11,12,13,14,19]. Cadmium chloride activates ERK and JNK MAPKs, which result in changes in the expression of *Bax* and *Bcl-2* [14,19]. The increased *Bax*/*Bcl-2* ratio acts pro-apoptotic, which is one of the pathways of apoptosis induction by cadmium compounds. However, it is worth mentioning that some reports demonstrate no significant effect of cadmium on *Bcl-2* and/or *Bax* expression [7,17,20,21], suggesting the involvement of other members of Bcl-2 protein family (for example Bcl-x_L_, Bid, Mcl-1) in cadmium-induced apoptosis [17,20,21].

## 4. Experimental Section

### 4.1. Reagents

THP-1 was obtained from American Type Culture Collection (ATCC, Rockville, MD, USA). RPMI medium, glutamine, and antibiotics (penicillin and streptomycin), cadmium chloride, phosphate buffered saline (PBS), dimethyl sulfoxide (DMSO) and Bradford reagent were purchased from Sigma–Aldrich (Poznań, Poland). Fetal bovine serum was purchased from Gibco (Gibco, Paisley, UK). Annexin V/fluorescein isothiocyanate (FITC) apoptosis detection kit was obtained from BD Pharmingen (San Jose, CA, USA). Fluorescence specific probe MitoSOX Red and JC-1 (5,5′,6,6′-tetrachloro-1,1′,3,3′-tetraethylbenzimidazol-carbocyane iodide) were purchased from Invitrogen, Warsaw, Poland.

### 4.2. Cell Culture and Treatment

The experiments were conducted on macrophages derived from a human monocytic cell line THP-1. THP-1 cell line was obtained originally as a transformed cell line from a patient with acute monocytic leukemia. The differentiation of THP-1 cells into macrophages was achieved by administration of 100nM phorbolmyristate acetate (PMA) and further incubation for 24 h. Differentiation of monocytes into THP-1 macrophages was confirmed by flow cytometry using anti-CD14 and anti-CD68 antibodies (monocytes are characterized by the presence of CD14 antibodies on their surface, and macrophages by CD68 antibodies). This procedure was performed in our preliminary study. The study showed that after 24 h from the addition of PMA to the cultures of THP-1 monocytes, they differentiate into adherent macrophages. The transformation events may result in different response profiles compared to non-transformed physiologically relevant macrophages. The adherent macrophages were washed three times with PBS and then incubated with cadmium chloride solutions for 48 h at 37 °C. The following concentrations of CdCl_2_ were used in this study: 5 nM, 20 nM, 200 nM and 2 μM. After 48 h, the cells were harvested by scraping and the pellets were obtained by centrifugation (800× *g*, 10 min). Afterwards, the cool PBS was added to the pellets and the samples were stored at −80 °C until the further analyses.

### 4.3. Fluorescence Studies

4.3.. Imaging of Mitochondrial Membrane Potential (ΔΨm)

Fluorescence studies were performed by specific probe (Invitrogen, Poznań, Poland) according to the procedure described by the manufacturer. JC-1 (5,5′,6,6′-tetrachloro-1,1′,3,3′-tetraethylbenzimidazol-carbocyane iodide) was used to measure mitochondrial membrane potential in the macrophages of control and Cd-treated cells.

JC-1 (10 mg) was dissolved in 1 mL DMSO and further diluted to the final concentration of 1 mg/mL in culture medium. JC-1 is a lipophilic cationic fluorochrome that exhibits potential-dependent accumulation in mitochondria and displays two colours of fluorescence: green (maximum emission at 510–527 nm with excitation at 490 nm) when the dye in the monomeric form accumulates in mitochondria with a low membrane potential (ΔΨm < 80–100 mV), and red-orange (maximum emission at 590 nm) in polarized mitochondria with membrane potential above 80–100 mV.

Macrophages were incubated with fluorochromein humidified 95% air/CO_2_ atmosphere at 37 °C for 30 min, and then washed with culture medium at room temperature. The preparations were examined under a confocal microscope Olympus FluoView 1000 (Olympus Polska sp. z o.o., Warsaw, Poland) using multiline Ar laser (for 458, 488 and 515 nm) and HeNe (Green) laser (for 543 nm).

#### 4.3.2. Quantitative Evaluation of Mitochondrial Membrane Potential (ΔΨm)

Cells were incubated with JC-1 at the same concentration and condition of microscopic studies [56] and washed three times with medium at room temperature. Fluorescence intensity was read in microplates for fluorescent studies (Eppendorf) by Asys UVM 340 microplate reader (ASYS Hitech GmbH, Eugendorf, Austria) at the wavelength specified above. Fluorescence was normalized to protein levels, measured by Bradford assay.

#### 4.3.3. Imaging of Mitochondrial ROS Generation

Mitochondrial superoxide level was visualized by fluorescent marker MitoSOX Red (Invitrogen) [56]. This fluorescent dye is highly selective for the detection of superoxide in living cell mitochondria. The reagent is oxidized by superoxide but not by other ROS or reactive nitrogen species, and exhibits red fluorescence (excitation 510 nm, emission 580 nm). MitoSOX Red stock solution (50 mM in DMSO) was diluted in the incubation medium to a final concentration of 2.5 µg/mL. Cells were incubated in this solution for 10 min (humidified 95% air/CO_2_ atmosphere at 37°C). Next, cells were washed with medium at room temperature and were examined under the confocal microscope Olympus FluoView 1000.

#### 4.3.4. Quantitative Evaluation of Mitochondrial ROS Generation

Cells were incubated with MitoSOX Red at the same concentration as for microscopic study, and then washed with medium at room temperature. Fluorescence was read by the microplate reader and normalized to protein levels, measured by Bradford assay.

#### 4.3.5. Imaging of Apoptosis Process

Macrophages, in the number of 5 × 10^5^ cells were incubated with cadmium chloride solutions according to the aforementioned procedure. After the end of cultivation, the cells were rinsed with PBS. Next, the cells were suspended in a binding buffer and stained with 1 ng/mL Annexin V-FITC (BD PharMingen, San Jose, CA, USA) and 5 ng/mL propidium iodide for 30 min in the dark. Cells that are viable are Annexin V-FITC and PI negative; cells that are in early apoptosis are Annexin V-FITC positive and PI negative; and cells that are in late apoptosis or already dead are both Annexin V-FITC and PI positive [57]. A dual-pass FITC/rhodamine filter set (Olympus Polska sp. z o.o., Warsaw, Poland) was applied. The preparations were examined under a fluorescence microscope (AxioObsrever.Z1, Carl Zeiss, Germany) using filter 09 (Filter set 09-487909-0000, Olympus Polska sp. z o.o., Warsaw, Poland) and a confocal microscope Olympus FluoView 1000.

### 4.4. Quantitative Evaluation of Apoptosis and Cell Viability by Flow Cytometry

Cells positive for apoptosis and necrosis were measured by Annexin V/propidium iodide assay (BD PharMingen San Jose, CA, USA). The externalization of phosphatydylserine as a marker of early-stage apoptosis was detected by the Annexin V protein conjugated to FITC (fluorescein isothiocyanate), whereas membrane damage due to late-stage apoptosis/necrosis was detected by binding of PI (propidium iodide) to nuclear DNA. After cadmium chloride treatment, cells were counted using a Burker hemocytometer (Abcam, Cambridge, UK) and then, 5 × 10^5^ cells were collected and washed twice with PBS. Next, the cells were suspended in a binding buffer and stained with 1 ng/mL Annexin V-FITC and 5 ng/mL propidium iodide for 30 min in the dark. Cells were analysed by Navios flow cytometer (Beckman Coulter, San Diego, CA, USA). Cells that are considered viable are FITC Annexin V and PI negative; cells that are in early apoptosis are FITC Annexin V positive and PI negative; and cells that are in late apoptosis or already dead are both FITC Annexin V and PI positive. Tests were performed in triplicate.

### 4.5. Quantitative Real-Time PCR Analysis (qRT-PCR) of Gene Expression

Quantitative analysis of mRNA expression of *Bcl-2* and *Bax* were performed in a two-step reverse transcription PCR. Total RNA was extracted from 50–100 mg tissue samples from cells using an RNeasy Lipid Tissue Mini Kit (Qiagen, Warsaw, Poland). After determination of the quantity and quality of isolated RNA using a NanoDrop ND-1000 spectrophotometer (NanoDrop Technologies, Wilmington, DE, USA), cDNA was prepared from 1 μg of total cellular RNA in 20 μL of reaction volume, using an Omniscript RT Kit (Qiagen, Warsaw, Poland) with oligo-dT primers, according to the manufacturer’s instructions.

Quantitative real-time PCR was performed using a CFX96 Real-Time PCR Detection System (Biorad, Warsaw, Poland). *GAPDH* was selected as a reference gene (the following primer pair was used for *GAPDH*: 5′-ATGACTCTACCCACGGCAAG-3′ and 5′-CTGGAAGATGGTGATGGGTT-3′). *Bcl-2* and *Bax* were analyzed by SYBR Green-based method: The 25-μL reaction mixture contained 12.5 μL of iQ SYBR Green Supermix (Biorad, Warsaw, Poland), 1.5 μL of cDNA, and one of the primer pairs (5′-GCCGGTTCAGGTACTCAGTCAT-3′ and 5′-CATGTGTGTGGAGAGCGTCAA-3′ for *Bcl2*; 5′-GTTGCCGTCAGAAAACATGTC-3′ and 5′-GCCGCCGTGGACACA-3′ for *Bax.* Every sample was analyzed simultaneously in two technical replicates, and mean *C*_t_ values were used for further investigation. Calculations were performed by ΔΔ*C*_t_ relative quantification method. The thresholds were set manually to compare data between runs and *C*_t_ values were extracted. All *C*_t_ values for each sample were normalized to the value obtained for *GAPDH*, the endogenous control gene. Fold change between groups was calculated from the means of the logarithmic expression values. Mean *C*_t_ values were: 18.4 for *GAPDH*, 22.5 for *Bax*, and 26.8 for *Bcl-2*.

### 4.6. Statistical Analysis

The statistical analysis of obtained results was conducted using Statistica 10 software (Statsoft, Warsaw, Poland). The results were expressed as arithmetical mean ± standard deviation (SD). Since the distribution in most cases deviated from normal (Shapiro-Wilk test), non-parametric tests were used. For related samples the significance was first checked with Friedmann’s ANOVA, and then significant results were subjected to the Wilcoxon matched-pair test. The level of significance was set at *p* < 0.05.

## 5. Conclusions

Our experiments demonstrate that cadmium, even at low environmental concentrations, exerts mitochondrial toxicity in THP-1 macrophages. Forty-eight-hour exposure of macrophages to very low cadmium concentrations reduces cell viability and results in cell death by apoptosis and necrosis. The decrease in mitochondrial membrane potential, increased ROS production, increased *Bax* and decreased *Bcl-2* mRNA expression are mitochondrial events involved in cadmium-induced apoptosis. Despite the fact that cadmium-induced alterations of the determinants were in fact small (although statistically significant), one may not exclude the cumulative effect of cadmium after long exposure periods. Due to high cadmium toxicity, efforts aiming at the reduction of its emissions to the environment are required.
